# Complete mitochondrial genome sequencing and preliminary phylogenetic analysis of *Chromis notata*

**DOI:** 10.1080/23802359.2021.1987175

**Published:** 2022-04-20

**Authors:** Ju-Hyung Jeon, Hyun-Hye Son, Seung-Soo Joo, Hyung-Joo Jin, Deuk-Hee Jin

**Affiliations:** Department of Marine Molecular Bioscience, Gangneung-Wonju National University, Gangneung, Korea

**Keywords:** *Chromis notata*, mitochondrial genome, phylogenetic analysis

## Abstract

*Chromis notata* (Temminck and Schlegel, 1843), commonly known as the pearl-spot chromis, is a damselfish that inhabits the northwestern region of the Pacific Ocean and the East China Sea. Interestingly, *C. notata* has been found to have morphological variations depending on the geographical area of collection. However, because there are insufficient molecular studies on *C. notata*, in this study, we determined its complete mitochondrial genome using PCR and phylogenetic analyses. The mitochondrial genome of *C. notata* was found to be 16,600 bp long, which consisted of 22 tRNA genes, 2 rRNA genes, 13 protein-coding genes, and 1 control region (D-loop). The base composition was 27.6% A, 24.8% T, 31.0% C, and 16.6% G. A phylogenetic tree reconstructed with the neighbor-joining method depicted a clone relationship with seven species of family Pomacentridae and our previous study based on *CO1* gene sequences. The complete mitochondrial genome is a valuable resource in classifying and conserving *C. notata*.

Fish belonging to the genus *Chromis* are widely distributed in tropical, subtropical, and temperate waters (Allen 1991; Randall and McCosker 1992). In Korea, three species of the genus *Chromis* (*Chromis notata, Chromis analis,* and *Chromis fumea*) have been identified (Koh et al. 1997), of which *C. notata* is the most commercially valuable. *C. notata*, commonly known as the pearl-spot *Chromis*, is distinguished by its large scales and dark brown body, and is characterized by its tendency to stay in one area for its entire lifespan. Although it is a small fish, it is famous as the representative fish species of Jeju Island. It inhabits the northwestern region of the Pacific Ocean and the East China Sea as well as coral reefs in the South Sea of Korea, the South China Sea, and the central subregion of Japan. Also, in coastal seas with 20 ∼ 30 m deep coral reefs, more than 100 individuals live in groups as colonies (Wantiez and Thollot 2000). Despite this, there are insufficient molecular studies on *C. notata* and therefore, in this study, we determined the complete mitochondrial genome to investigate the genetic characteristics of *C. notata* inhabiting Korea. Specimens were collected at Pier 2 in Jeju Island, Korea (N 33°31'09.0″, E 126°31'59.0"). The specimens and DNA are deposited at the Laboratory of Marine Molecular Biology, Gangneung-Wonju National University, Korea (voucher number: GWNU-MB12012, dhjin@gwnu.ac.kr). Long and accurate PCR (LA PCR) was performed to amplify the complete mitochondrial genome sequence using a set of universal primers (Inoue et al. 2001) as well as designed primers. Sequencing was conducted using the primer walking method on an ABI 3730XL DNA Analyzer (Applied Biosystems Inc., Foster City, CA, USA). The sequences were assembled, aligned, and annotated using MEGA6.0 (Tamura et al. 2013) and tRNAscan-SE 2.0 (Chan and Lowe 2019). The genome sequence and gene annotations of *C. notata* are deposited in GenBank under the accession number MT800513.

The complete mitochondrial genome of *C. notata* was similar to that of most other vertebrates (Inoue et al. 2000). It was 16,600 bp in length, consisting of 13 protein-coding genes (PCGs), 22 tRNA genes, 2 rRNA genes and one control region (CR). Except for the NADH dehydrogenase subunit 6 (*ND6*) and eight tRNA genes (Gln, Ala, Asn, Cys, Tyr, Ser, Glu, and Pro), the remaining 28 genes were encoded on the heavy strand (H-strand). The AT content of the mitochondrial genome and PCGs was 52.4 and 51.5%, respectively. Of the 13 PCGs, only the cytochrome c oxidase 1 (*CO1*) gene started with the codon GTG, whereas the other PCGs started with the codon ATG. The 22 tRNA genes folded into typical cloverleaf secondary structures and ranged between 65 and 79 bp. The 878 bp-long CR was located between the tRNA-Phe and tRNA-Pro genes. The phylogenetic relationship was analyzed with seven species of family Pomacentridae and our previous study (Sim et al. 2018). The neighbor-joining method was used to reconstruct the phylogenetic tree based on the *CO1* partial gene sequences (408 bp). The results of the phylogenetic analysis demonstrated that *C. notata* is closely related to *Chromis fumea* (KU944347.1) and *Chromis nitida* (HQ956578.1) (Figure 1).

In Korea, the phylogenetic relationship of Chrominae subfamily is not well known (Koh and Park 2007). Additionally, the data in the genus *Chromis* are not sufficient to proceed with molecular analyses, which is why researchers have no choice but to rely on morphological analysis. However, *C. notata* has morphological variations depending on the geographical area of collection. Some of these morphological variations include changes in the black pattern at the base of the pectoral fin from triangular to circular, and changes in the length of the badge fin to become shorter, in geographical regions further north from Jeju Island (Kim and Kim 1997). To study these variations, the determination of the mitochondrial genome of *C. notata* is complementary to further molecular research. Even though the results of this analysis only include one species of the genus *Chromis*, the mitochondrial genomic data of *C. notata* will provide useful information for future research involving other species of the genus *Chromis*.

**Figure 1.**
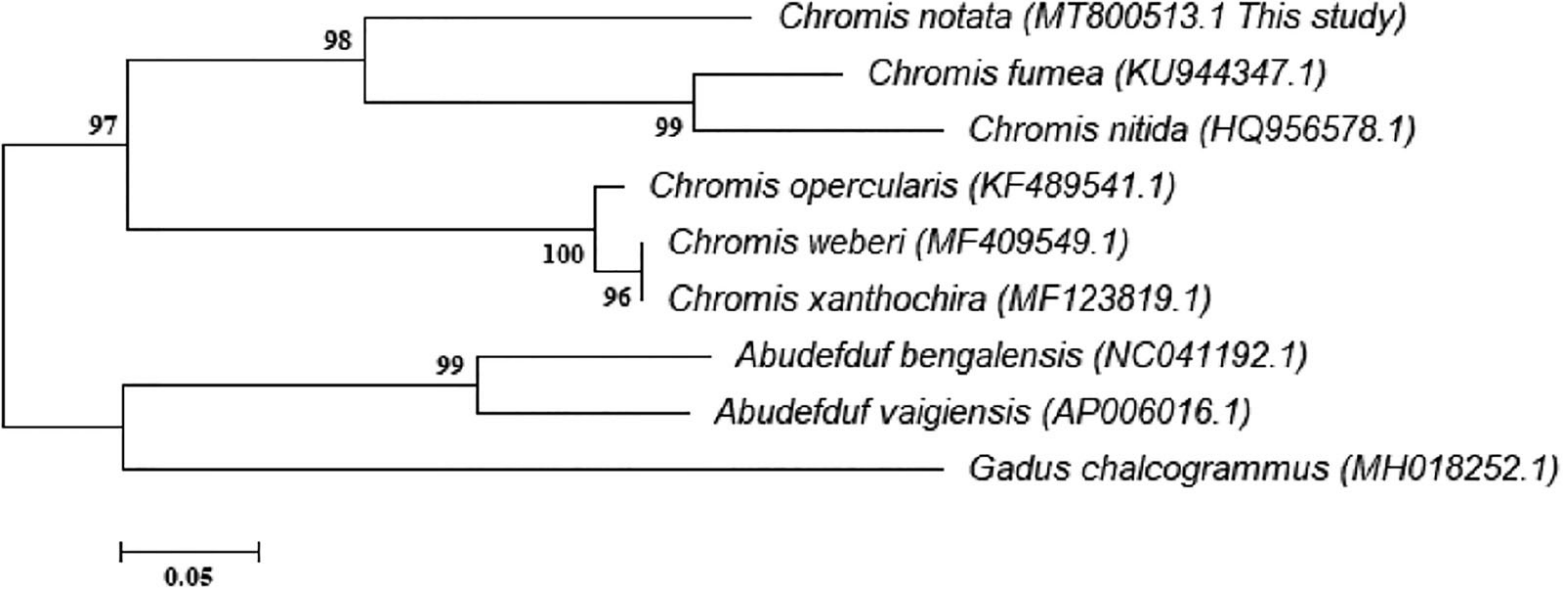
The neighbour-joining phylogenetic tree was constructed with MEGA 6 using the CO1 partial gene sequence. The gene tree of *Chromis notata*, along with seven of family Pomacentridae and our previous study (Sim et al. 2018) based on mitochondrial CO1 partial gene sequences, was analyzed by NJ method. Bootstrap replicates were conducted 10,000 times.

## Data Availability

The genome sequence data that support the findings of this study are openly available in GenBank of NCBI at (https://www.ncbi.nlm.nih.gov/) under the accession number of MT800513.
